# An optimized method for ^15^N R_1_ relaxation rate measurements in non-deuterated proteins

**DOI:** 10.1007/s10858-015-9937-4

**Published:** 2015-05-07

**Authors:** Margarida Gairí, Andrey Dyachenko, M. Teresa González, Miguel Feliz, Miquel Pons, Ernest Giralt

**Affiliations:** NMR Facility, Scientific and Technological Centers, University of Barcelona (CCiTUB), Baldiri Reixac 10, 08028 Barcelona, Spain; Institute for Research in Biomedicine (IRB), Baldiri Reixac 10, 08028 Barcelona, Spain; Biomolecular NMR Laboratory and Organic Chemistry Department, University of Barcelona, Baldiri Reixac 10, 08028 Barcelona, Spain

**Keywords:** ^15^N relaxation, Longitudinal relaxation, R_1_, Water saturation, ^15^N CSA/^1^H–^15^N dipolar cross-correlated relaxation (CC), Radiation damping (RD)

## Abstract

^15^N longitudinal relaxation rates are extensively used for the characterization of protein dynamics; however, their accurate measurement is hindered by systematic errors. ^15^N CSA/^1^H–^15^N dipolar cross-correlated relaxation (CC) and amide proton exchange saturation transfer from water protons are the two main sources of systematic errors in the determination of ^15^N R_1_ rates through ^1^H–^15^N HSQC-based experiments. CC is usually suppressed through a train of 180° proton pulses applied during the variable ^15^N relaxation period (T), which can perturb water magnetization. Thus CC cancellation is required in such a way as to minimize water saturation effects. Here we examined the level of water saturation during the T period caused by various types of inversion proton pulses to suppress CC: (I) amide-selective IBURP-2; (II) cosine-modulated IBURP-2; (III) Watergate-like blocks; and (IV) non-selective hard. We additionally demonstrate the effect of uncontrolled saturation of aliphatic protons on ^15^N R_1_ rates. In this paper we present an optimized pulse sequence that takes into account the crucial effect of controlling also the saturation of the aliphatic protons during ^15^N R_1_ measurements in non-deuterated proteins. We show that using cosine-modulated IBURP-2 pulses spaced 40 ms to cancel CC in this optimized pulse program is the method of choice to minimize systematic errors coming from water and aliphatic protons saturation effects.

**Electronic supplementary material:**

The online version of this article (doi:10.1007/s10858-015-9937-4) contains supplementary material, which is available to authorized users.

## Introduction

The intrinsic dynamic properties of proteins play a key role in their function. Information about dynamics on several timescales can be studied through solution NMR spectroscopy using ^15^N spin relaxation experiments on ^15^N-labeled protein samples. Longitudinal relaxation rate R_1_, transverse relaxation rate R_2_, and ^15^N–^1^H steady-state NOE parameters of backbone ^15^N nuclei measured at various magnetic fields are commonly used to address global and local protein dynamics at ps–ns and μs–ms timescales (Torchia 2011).

Quantitative analysis of backbone dynamics requires accurate and consistent relaxation measurements (Morin 2011). It has long been recognized that systematic errors in the measurements of ^15^N relaxation rates R_1_, R_2_ and ^15^N–^1^H NOE can be significant and far larger than random errors. Two main sources of these errors have been identified as amide proton exchange saturation transfer from water protons (Grzesiek and Bax 1993; Farrow et al. 1994; Ferrage et al. 2010; Chen and Tjandra 2011; Lakomek et al. 2012; Jurt and Zerbe 2012) on the one hand, and ^15^N CSA/^1^H–^15^N dipolar cross-correlated (CC) relaxation (Boyd et al. 1990; Kay et al. 1992a; Palmer et al. 1992; Kumar et al. 2000; Gong and Ishima 2007; Ferrage et al. 2009; Ishima 2014) on the other.

Water saturation has a strong effect on ^15^N R_1_ measurements (Chen and Tjandra 2011). It can arise as a consequence of improper handling of water magnetization effects of RF pulses and/or pulsed field gradients (PFG) during the whole pulse program (Stonehouse et al. 1994). Saturation can be transferred through proton–proton exchange or via NOE to exchangeable amide protons—directly detected in HSQC-based R_1_ measurements—during the inter-scan delay and it distorts proton amide signal intensities. In addition, varying degrees of water saturation during variable ^15^N magnetization decay periods can alter the intensity decay, thus causing systematic errors in R_1_ relaxation rates (Lakomek et al. 2012). Cryogenic probeheads (Kovacs et al. 2005) exacerbate water suppression-related problems by strong radiation damping (RD) effects (Krishnan and Murali 2013) especially at high magnetic fields (Shishmarev and Otting 2011).

Another important source of systematic errors on R_1_ measurements is longitudinal CC between ^1^H–^15^N DD and ^15^N CSA. The CSA component increases also with magnetic fields. Insufficient cancellation of cross-correlation effects at very high fields is reported to generate significant deviations in ^15^N R_1_ rates for deuterated proteins (Ishima 2014). CC is typically suppressed by applying a series of proton 180° pulses during the variable ^15^N relaxation period (Kay et al. 1992a). However, these pulses can perturb water magnetization if care is not taken to prevent it. In addition, they can cause unwanted saturation effects on protein protons. In particular, saturation of aliphatic protons in non-deuterated proteins may cause significant errors on ^15^N R_1_ measurements, a phenomenon that has not been properly addressed so far.

In this paper we present an optimized method to determine ^15^N R_1_ rates on non-deuterated proteins, which can be used at high fields using cryogenic probes, in order to address the problems described above. The optimized sequence relies on the control of both water and aliphatic protons saturation while CC-suppressing pulses are applied during the variable ^15^N relaxation period.

## Experimental section

Our study was carried out with a fully protonated ^15^N-labeled sample of the small and highly stable third Igg-binding domain of protein G (GB3).

The NMR sample consisted of 1.5 mM ^15^N-labeled GB3 in 25 mM phosphate buffer, 25 mM NaCl, 0.02 % NaN_3_ and 10 % D_2_O, pH 6.5. ^15^N longitudinal relaxation experiments were performed on a 600 MHz Bruker Avance III and an 800 MHz Bruker DRX NMR spectrometer operating at 60.79 and 81.06 MHz ^15^N resonance frequencies, respectively. Both instruments were equipped with TCI cryogenic probes and a Z-gradient coil. Data were recorded at 298 K.

**Fig. 1:**
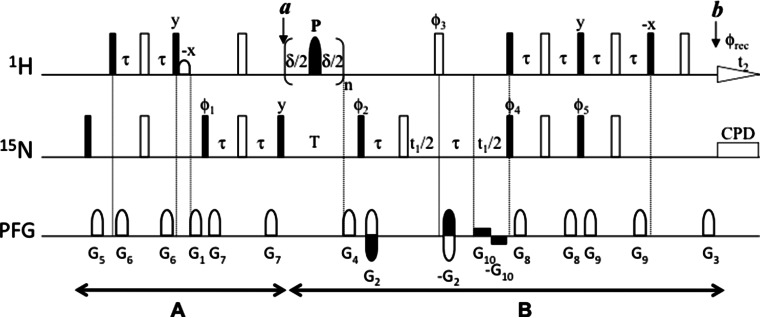
Pulse program for measuring ^15^N R_1_ relaxation rates in non-deuterated proteins, through a ^1^H–^15^N HSQC-based experiment. *Narrow filled rectangles* and *open rectangles* correspond to 90° and 180° flip angle pulses, respectively, with phase x unless indicated. The *open bell* in proton channel corresponds to a 90° sinc-shaped low-power water-selective pulse, a (sinx)/x function, with a duration of 1 ms. The *filled bells* (*P*) applied to proton during the variable ^15^N relaxation delay T correspond to 180° pulses to cancel CC during this period. They are applied as a train of pulses spaced by intervals of duration δ, in such a way that the loop is repeated an even number of times. The number of 180° pulses (n) is varied to yield different ^15^N relaxation delays (T = 2 × n × δ). Several types of 180° inversion elements (*P*) have been used to cancel CC in different R_1_ experiments: (I) off-resonance selective amide proton IBURP-2 pulses (1.9 ms duration, offset = 2400 Hz at 600 MHz; 1.5 ms, offset = 3000 Hz at 800 MHz); (II) cosine modulated IBURP-2 pulses (1.9 ms duration, offset = ±2400 Hz at 600 MHz; 1.5 ms, offset = ±3000 Hz at 800 MHz); (III) Watergate-like block consisting of three pulses: water-selective low-power 90° pulse (1 ms, *square shaped pulse*)—180° hard pulse (20–24 μs length at high power)—water-selective low-power 90° pulse (1 ms, *square shaped pulse*); (IV) non-selective hard pulses (20–24 μs at high power). Inter-space delays δ = 5 ms (pulsing rate R_p_ = 200 s^−1^) or δ = 40 ms (pulsing rate R_p_ = 25 s^−1^) were used. τ = 2.78 ms. All PFG are along z-axis, smoothed-square shaped. Duration and strength are indicated in *parenthesis*: G1 (1 ms; 25 G/cm), G2 (1 ms; 40 G/cm), G3 (1 ms; 8.1 G/cm), G4 (1 ms; −25 G/cm at 600 MHz or 11.5 G/cm at 800 MHz), G5 (1 ms; 4.5 G/cm), G6 (1 ms; 8.5 G/cm), G7 (0.5 ms; 15.5 G/cm), G8 (0.5 ms; 5.5 G/cm), G9 (0.5 ms; 6.5 G/cm), G10 (0.6 G/cm). Phase cycling: Φ_1_ = 4(x), 4(−x); Φ_2_ = y, −y; Φ_3_ = 2(x), 2(−x); Φ_4_ = 2(x), 2(−x); Φ_5_ = 2(y), 2(−y); Φ_rec_ = x, −x, x, x, −x, x, x, −x. Quadrature detection in F1 is implemented using the gradient-enhanced echo/anti-echo scheme (Kay et al. 1992b) inverting the polarity of PFG G2 and also the phase Φ_5_ for the second FID. ^15^N GARP decoupling sequences (Shaka and Keeler 1987) were applied during ^1^H acquisition at a field strength of around 1 kHz

We used 1D versions of the pulse scheme shown in Fig. 1 to measure the water signal intensity—reporting the level of non-saturated water (M_*nsw*_)—using a short angle readout pulse. Readout pulses were introduced just before the ^15^N relaxation period T (point *a*) and at the start of the regular ^1^H acquisition time (point *b*). Experiments were carried out at 600 and 800 MHz. Simultaneous phase cycling (0°, 180°) of the receiver and the readout pulse allowed the cancelation of the protein signal. Readout pulses shorter than 1° were used in order to avoid artifacts caused by RD. The resulting water signal was integrated (I^x^) in the region 6.5–2.9 ppm (spectral width of 3.65 ppm which corresponds to a range equal to 50 times the observed full peak width at half maximum). Water saturation is given with respect to the signal intensity measured following a single short angle proton pulse (I^ref^). The water saturation level (M_*sw*_) was calculated as:

$$ {\text{M}}_{\text{sw}}^{\text{x}} = \left( {1 - {\text{M}}_{\text{nsw}}^{\text{x}} } \right) \times 100 = \left( {1 - \frac{{{\text{I}}^{\text{x}} }}{{{\text{I}}^{\text{ref}} }}} \right) \times 100 $$

where I^x^ is the signal intensity at point x = *a* or *b* and I^ref^ is the intensity of the reference signal.

The relative water saturation at point *b* with respect to point *a*, as a percentage, is given by:

$$ {\text{M}}_{\text{sw}}^{\text{b/a}} = \left( {\frac{{{\text{M}}_{\text{nsw}}^{\text{a}} - {\text{M}}_{\text{nsw}}^{\text{b}} }}{{{\text{M}}_{\text{nsw}}^{\text{a}} }}} \right) \times 100 = \left( {\frac{{{\text{I}}^{\text{a}} - {\text{I}}^{\text{b}} }}{{{\text{I}}^{\text{a}} }}} \right) \times 100 $$

**Fig. 2:**
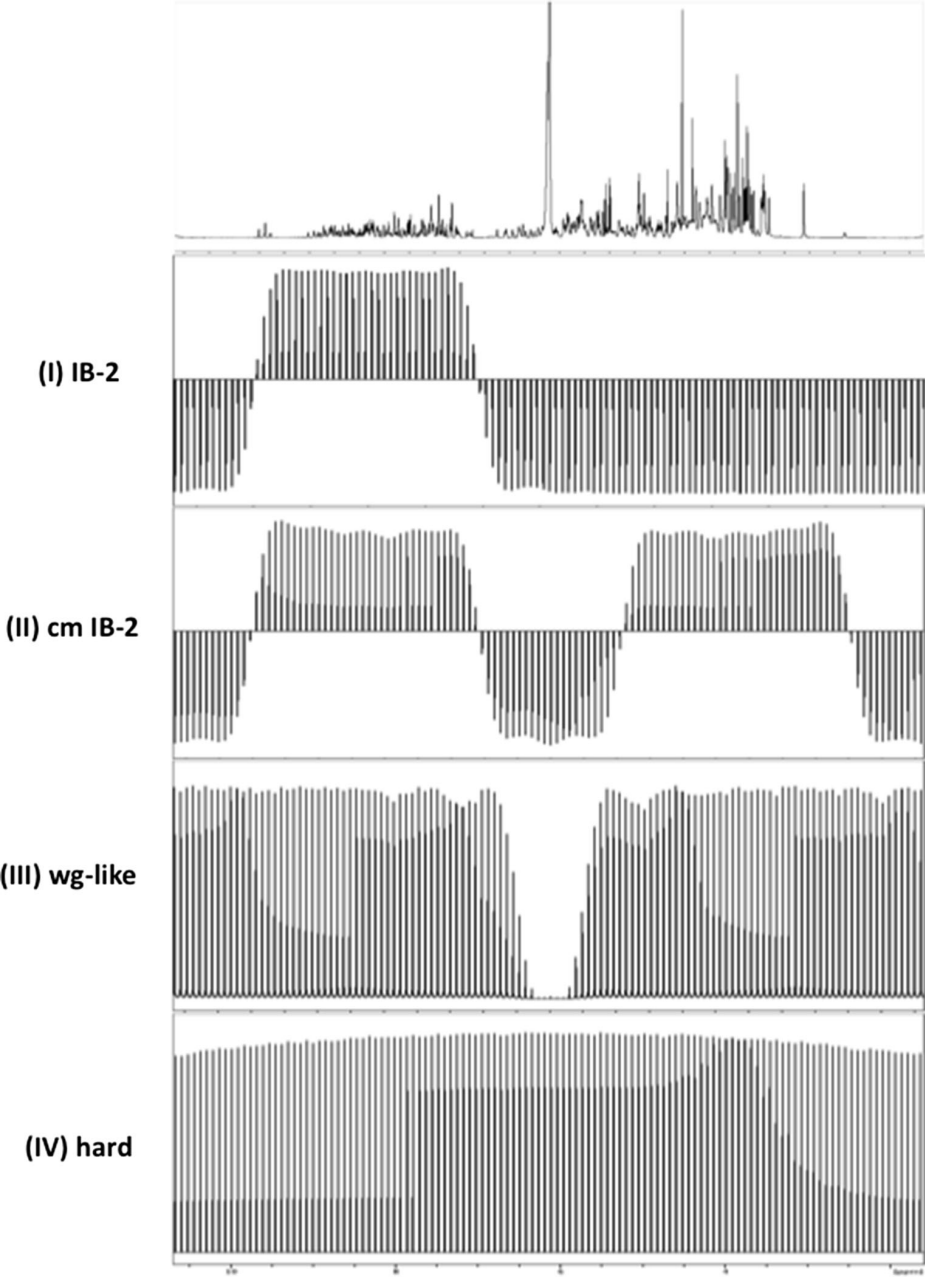
^1^H spectrum of ^15^N–GB3 and experimental excitation profiles at 800 MHz for several types of proton inversion elements used to cancel CC during the ^15^N relaxation period T: (I) IB-2: amide-selective IBURP-2 pulse (1.5 ms, offset 3000 Hz from water frequency); (II) cm IB-2: cosine-modulated IBURP-2 pulse (1.5 ms, offset ± 3000 Hz from water frequency); (III) wg-like: Watergate-like pulses: water-selective 90°_−x_ pulse (1 ms, *square*)—hard 180°_x_ pulse (24 μs)—water-selective 90°_−x_ pulse (1 ms, *square*); (IV) hard: non-selective hard 180° pulse (24 μs). Excitation profiles for I, II and IV were measured with a pulse program consisting of the selective 180° pulse, followed by a PFG and a final short readout pulse, while profile for scheme III was generated using a pulse program consisting of an initial 90° pulse, followed by a 90°_sel_–180°_hard_–90°_sel_ block inserted in a PFG echo and a final shord readout pulse. Each spectrum was acquired with a single scan and the offsets vary along 12,000 Hz in 100 Hz-steps. A CuSO_4_-doped water sample in D_2_O was used

1D experiments were performed using fixed ^15^N relaxation periods of 80 and 800 ms. The first t_1_ increment (10–12 μs) was measured in all cases. Saturated water was evaluated for several approaches to cancel CC, so the following 180° proton pulses (P) types were tested during T (Fig. 2): (I) a train of off-resonance amide-selective IBURP-2 pulses (Geen and Freeman 1991) (1.9 ms length, offset 2400 Hz with respect to water frequency, at 600 MHz; 1.5 ms length, offset of 3000 Hz with respect to water frequency, at 800 MHz); (II) a train of cosine-modulated off-resonance IBURP-2 pulses which selectively invert two spectral ranges simultaneously, centered at +2400 and −2400 Hz (+3000 and −3000 Hz) with respect to the water frequency at 600 MHz (800 MHz), pulse lengths were 1.9 and 1.5 ms at 600 and 800 MHz, respectively, power levels were increased by 6 dB with respect to those used for IBURP-2 pulses to account for the second frequency component; (III) a train of Watergate-like (Piotto et al. 1992) blocks, each one consisting of a 90° water-selective pulse (1 ms, square pulse) followed by a 180° hard pulse (20–24 μs) and after by another 90° water-selective pulse (1 ms, square pulse) and (IV) a train of hard 180° pulses (20–24 μs).

**Fig. 3:**
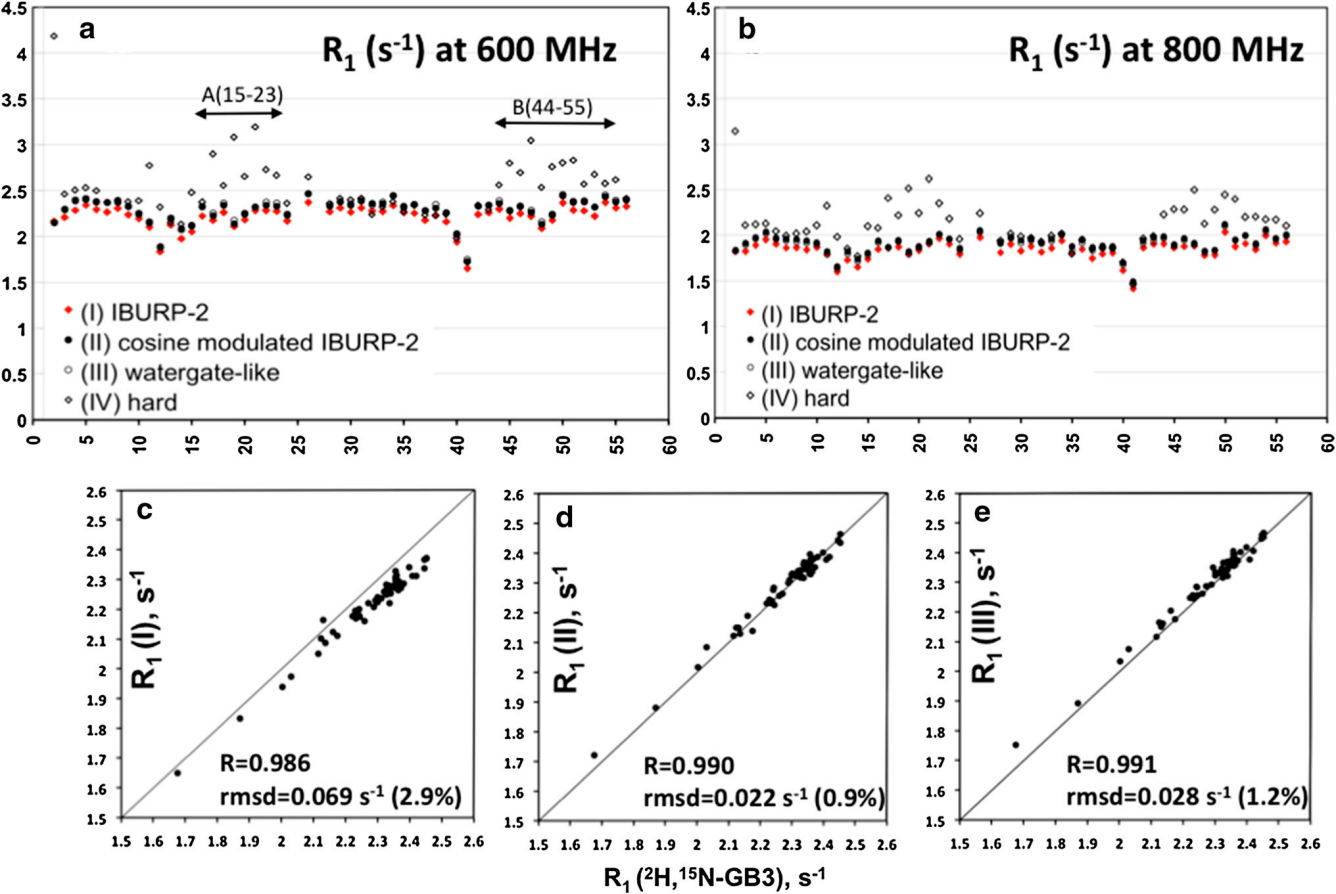
^15^N R_1_ relaxation rates measured at 600 MHz (**a**) and 800 MHz (**b**) for non-deuterated ^15^N–GB3 using several CC-suppressing schemes during the variable ^15^N relaxation delay T. A proton pulsing rate R_p_ = 25 s^−1^ (180° pulses *P* spaced δ = 40 ms, see Fig. 1) was used in all cases. **a**, **b** R_1_ rates measured using IBURP-2 pulses (I), cosine-modulated IBURP-2 (II), Watergate-like pulses (III) or hard pulses (IV) for suppressing CC; **c** correlation plot of R_1_ rates measured at 600 MHz with approach (I) and with a deuterated GB3 sample (Lakomek et al. 2012); **d** correlation plot of R_1_ rates measured at 600 MHz with approach (II) and with deuterated GB3; **e** correlation plot of R_1_ rates measured at 600 MHz with approach (III) and with deuterated GB3

HSQC-based R_1_ experiments were performed with the pulse sequence shown in Fig. 1 using the previously described CC-suppressing approaches. R_1_ rates at 600 and 800 MHz are shown in Fig. 3. The spectral widths for experiments carried out at 600 MHz were set to 8418 Hz (F2) and 2189 Hz (F1) for ^1^H and ^15^N, respectively, with sampling durations of 107 ms (t_2_) and 58 ms (t_1_). For experiments measured at 800 MHz, spectral widths of 11,160 Hz (F2) and 2838 Hz (F1) for ^1^H and ^15^N, respectively, and sampling durations of 67 ms (t_2_) and 53 ms (t_1_) were used. Radio-frequency carriers were set to 4.7 ppm for ^1^H and 118 ppm for ^15^N. For each relaxation time measurement, a series of eight 2D experiments with ^15^N relaxation delays T ranging from 0 to 800 ms (0, 80, 160, 240, 320, 400, 560, 800 ms) were collected in a randomized order. Eight scans per FID were recorded, and a recycle delay between scans of 1.7 s was used.

**Fig. 4:**
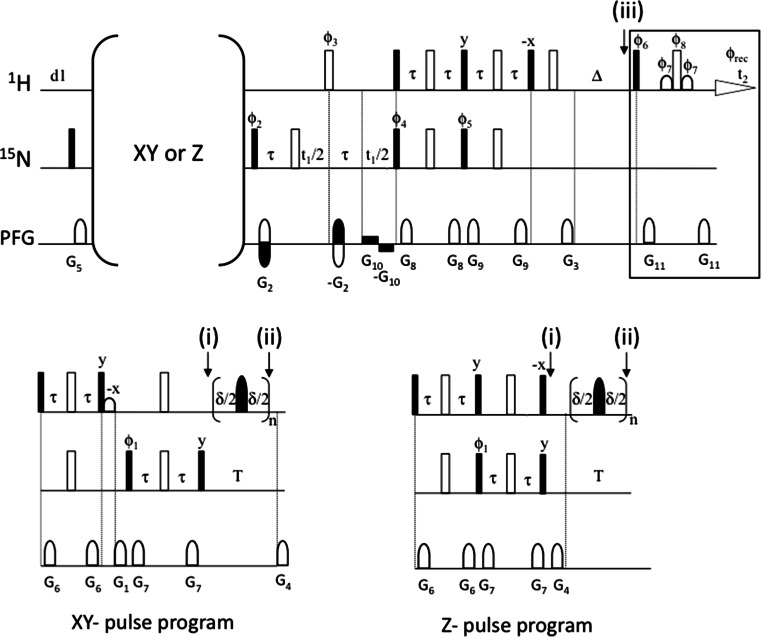
Pulse programs (XY and Z) to evaluate the amount of H^R^ and H^N^ polarization present at different points in the sequence used to determine ^15^N R_1_ relaxation rates. A 90° readout pulse followed by a Watergate block (*boxed*) was applied in either point (i), (ii) or (iii). Δ corresponds to the recycle delay used in R_1_ measurements. Therefore, point (iii) corresponds to the start of the scans under steady-state conditions. The first t_1_ increment (12 μs) was acquired in the 1D versions of the pulse program. In the XY-pulse scheme the aliphatic protons magnetization remains in the transverse plane and is saturated by the PFG G1 applied before the T period. In the Z-pulse sequence the aliphatic protons magnetization is aligned along the z-axis at the start of this period. The same pulses, delays and PFG as those described in Fig. 1 were used. Phase cycling: Φ_1_ = 4(x), 4(−x); Φ_2_ = y, −y; Φ_3_ = 2(x), 2(−x); Φ_4_ = 2(x), 2(−x); Φ_5_ = 2(y), 2(−y); Φ_6_ = x, −x; Φ_7_ = 2(x), 2(y), 2(−x), 2(−y); Φ_8_ = 2(−x), 2(−y), 2(x), 2(y); Φ_rec_ = x, −x, x, x

**Fig. 5:**
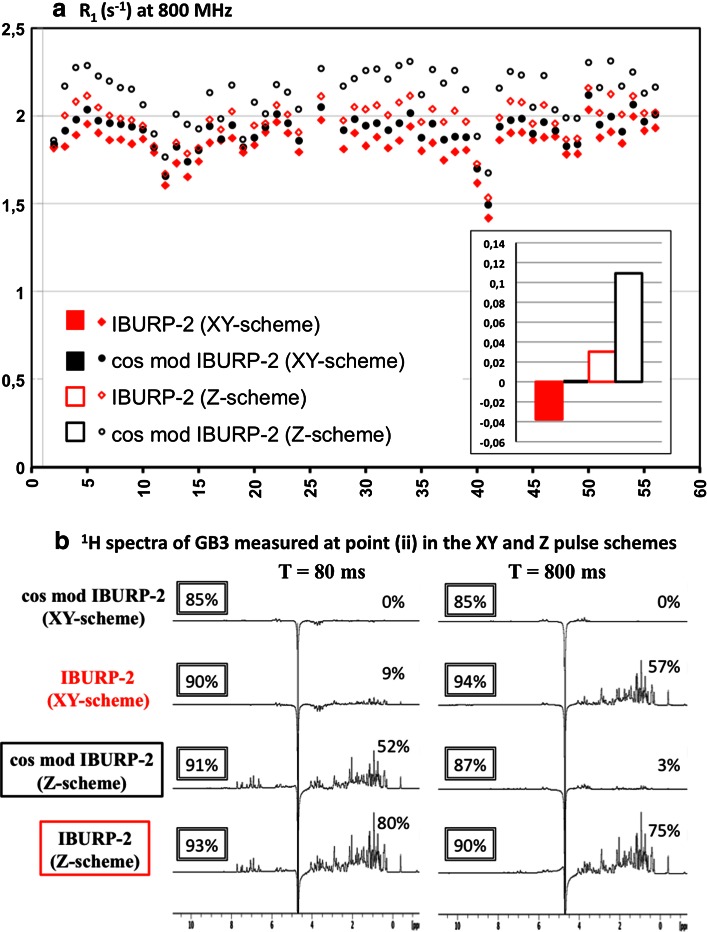
**a** ^15^N R_1_ relaxation rates measured at 800 MHz for non-deuterated ^15^N–GB3 using XY- and Z-pulse schemes (see Fig. 4), and CC-suppressing schemes based on amide-selective IBURP-2 pulses or cosine-modulated IBURP-2 pulses (R_p_ = 25 s^−1^), applied during the variable ^15^N relaxation delay (T). Deviations on average R_1_ rates respect to those obtained with the XY-sequence and cosine-modulated IBURP-2 pulses, (<R_1_> − <R_1_>_XYcmIB2_)/<R_1_>_XYcmIB2_ are shown inside the *box*. **b** ^1^H spectra of GB3 at 800 MHz measured using the XY and Z-pulse schemes in Fig. 4. The spectra show the amount of non-saturated H^N^ and H^R^ magnetization at point (ii), measured immediately after the application of a train of amide-selective IBURP-2 pulses or cosine-modulated IBURP-2 pulses (with 40 ms inter-pulse spacing) for relaxation periods T of 80 and 800 ms. The relative integral of the aliphatic region between 2.25 and 0.25 ppm is given in the *right* part of each spectrum. The relative integral of the amide region between 9.55 and 7.25 ppm measured at point (iii) with Δ = 1.7 s is given inside the *boxes* at the *left* part of the spectra. Percentages are calculated respect to the amount of the equilibrium H^R^ or H^N^ polarization measured at point (iii) for Δ = 3.5 s in the corresponding sequence

The aliphatic proton magnetization at the beginning of the variable ^15^N relaxation period in the pulse sequence of Fig. 1 is dephased in the xy-plane (XY-pulse program). Alternative sequences for measuring ^15^N R_1_ rates have this magnetization on the z-axis (Z-pulse program). The 1D versions of the XY- and Z-sequences (Fig. 4) were used to evaluate the amount of amide proton (H^N^) and aliphatic proton (H^R^) polarization at several points of the pulse scheme. Longitudinal proton magnetization was measured with a 90° readout pulse followed by a Watergate block for water suppression (see box in Fig. 4) in the following points: (i) just before the ^15^N relaxation period (T); (ii) just after this period T, for T = 80 and T = 800 ms, and with amide-selective IBURP-2 pulses spaced 40 ms or cosine-modulated IBURP-2 pulses spaced 40 ms as inversion elements to cancel CC; (iii) at the final point of the pulse program after a delay (Δ) added to allow for proton magnetization to relax to equilibrium (+z) for a time period identical to the recycle delay in the pulse scheme used to measure ^15^N R_1_. In this last case, two values of Δ were evaluated: 1.7 and 3.5 s. Thus, proton magnetization at point (iii) represents the amount of polarization at the beginning of the sequence under steady-state conditions. Experiments were performed at 800 MHz. The first t_1_ increment (12 μs) was acquired. Eight scans were measured and a recycle delay d1 = 10 s was used. H^N^ and H^R^ polarization were evaluated by relative integration of the amide region between 9.55 and 7.25 ppm and relative integration of the aliphatic region between 2.25 and 0.25 ppm, respectively, in the corresponding GB3 ^1^H spectra (see Fig. 5). Relative integrations were calculated respect to the amount of the equilibrium H^N^ or H^R^ polarization measured at point (iii) for Δ = 3.5 s in the corresponding sequence.

^15^N R_1_ rates measurements performed with Z-pulse program at 800 MHz were carried out in the same conditions as previously described for XY-pulse scheme (Fig. 1).

All spectra were processed with Topspin from Bruker, and relaxation time constants and fitting errors were extracted using the free software R programming language (www.r-project.org).

## Results and discussion

### Exploring the effect of water saturation caused by CC-suppressing pulses on ^15^N R_1_ measurements

Two sources of non-equilibrium water magnetization can be considered in experiments for measuring ^15^N R_1_ relaxation in proteins: partially saturated water coming from water magnetization handling before the variable ^15^N relaxation period (Chen and Tjandra 2011) on one hand, and additional water saturation created as a side effect of the 180° proton pulses used to cancel CC effects during this period (Lakomek et al. 2012) on the other.

Preventing water saturation while efficiently suppressing CC is crucial for accurately measuring R_1_ rates. The pulse scheme depicted in Fig. 1 is designed for this purpose: PFG around 180° pulses during spin-echoes when water is transverse minimize RD; a water-flip-back pulse during the initial INEPT keeps water at +z axis; water magnetization is brought to +z at the start of ^15^N relaxation period T; bipolar PFG are used during the second part of t_1_ evolution time where water remains at −z axis to reduce RD; and PFG are immediately applied to minimize RD every time water is placed at −z axis.

The pulse sequence in Fig. 1 can be divided into two blocks, A and B. Ideally, water magnetization should reach the +z axis just after each of these modules (points *a*, *b*) and retain equilibrium magnetization (no saturation). However, as a consequence of imperfections of RF pulses, water dephasing and rephasing caused by PFG, and strong RD effects present at high fields with cryoprobes, the path for water magnetization deviates from this ideal situation and is partially saturated. By comparing water saturation at points *a* and *b* in the pulse sequence, we can evaluate the additional water saturation introduced in block B, which includes mostly the effect of the CC-suppression scheme. Since CC-suppression is applied for a variable duration, water saturation can modify the intensity of amide proton decay differently for each value of T and introduce systematic errors in the measurement of ^15^N R_1_ relaxation rates.

The most widely used approaches to suppress CC are: (i) amide-selective IBURP-2 pulses (Geen and Freeman 1991), inverting only magnetization of the amide protons (Lakomek et al. 2012; Chill et al. 2006); (ii) cosine-modulated selective pulses (Smallcombe 1993), exciting both the amide and the aliphatic region of the protein while performing zero excitation at the water resonance (Farrow et al. 1994; Zhu et al. 2000); and (iii) combination of selective and non-selective pulses in a Watergate-like (Piotto et al. 1992) way to selectively invert non-water protons (Chen and Tjandra 2011).

Here we evaluated the water saturation level caused by the three CC-suppressing schemes described above using a 1D version of the pulse program depicted in Fig. 1. Non-selective hard 180° proton pulses were also included in our study as a reference. Experiments were carried out at 800 and 600 MHz, using TCI cryoprobes with the following proton pulses: amide-selective 180° off-resonance IBURP-2 pulses (I); cosine-modulated 180° IBURP-2 pulses (II); Watergate scheme based on the block 90° water-selective pulse—180° hard pulse—90° water-selective pulse (III); and non-selective 180° hard pulses (IV). Figure 2 shows the corresponding experimental excitation profiles at 800 MHz. Experimental profiles at 600 MHz are presented in Fig. S1 SM. Selective excitation schemes should minimally affect water magnetization in contrast to the use of hard pulses. However, residual water saturation present after the various schemes is significantly different, in practice.

Water saturation levels were measured after the application of the above mentioned CC-suppressing schemes for short (80 ms) and long (800 ms) ^15^N relaxation periods T. In addition, the effect of pulsing rate (R_p_) during cancellation of CC was also studied using two rates: fast pulsing regime (200 s^−1^, proton pulses spaced 5 ms) and slow pulsing regime (25 s^−1^, proton pulses spaced 40 ms). Results are presented in Table 1.

**Table 1:** Level of saturated water generated between the start of ^15^N relaxation delay (point *a*) and the start of proton acquisition period (point *b*) in the pulse program of Fig. 1

	(I) IB-2		(II) cm IB-2		(III) wg-like		(IV) hard	
	25 s^−1^	200 s^−1^	25 s^−1^	200 s^−1^	25 s^−1^	200 s^−1^	25 s^−1^	200 s^−1^
Degree of saturated water (%) at 600 MHz								
${\text{M}}_{\text{sw(80ms)}}^{\text{b/a}}$	10	14	10	13	11	25	25	90
${\text{M}}_{\text{sw(800ms)}}^{\text{b/a}}$	8	23	6	29	10	99	96	97
$\Delta {\text{M}}_{{{\text{sw(800}} - 8 0 )}}^{\text{b/a}}$	−2	9	−4	16	−1	74	71	7
Degree of saturated water (%) at 800 MHz								
${\text{M}}_{\text{sw(80ms)}}^{\text{b/a}}$	11	14	10	13	9	19	80	79
${\text{M}}_{\text{sw(800ms)}}^{\text{b/a}}$	8	23	7	26	10	63	97	99
$\Delta {\text{M}}_{{{\text{sw(800}} - 8 0 )}}^{\text{b/a}}$	−3	9	−3	13	1	44	17	20

Water saturation (M_sw_) is calculated, as described in “Experimental section”, for different schemes to suppress CC: (I) IB-2: a train of amide-selective IBURP-2 180° proton pulses; (II) cm IB-2: a train of cosine-modulated IBURP-2 180° proton pulses; (III) wg-like: a train of Watergate-like blocks consisting of 90° water-selective pulse—180° hard proton pulse—90° water-selective pulse; (IV) hard: a train of 180° hard proton pulses. Water saturation has been measured for two proton pulsing rates (R_p_), 25 and 200 s^−1^. For each one of the CC-suppressing schemes, two lengths of the ^15^N relaxation period (T), 80 and 800 ms, have been evaluated. $\Delta {\text{M}}_{{{\text{sw(800}} - 8 0 )}}^{\text{b/a}}$, calculated as the difference ${\text{M}}_{\text{sw(800ms)}}^{\text{b/a}} - {\text{M}}_{\text{sw(80ms)}}^{\text{b/a}}$, refers to the increment of water saturation occurred at long T periods. Results at 600 and 800 MHz are shown

The level of water saturation at point *a* was about 20 %, both at 800 and 600 MHz. The amount of saturated water generated between points *a* and *b*, ${\text{M}}_{\text{sw}}^{\text{b/a}}$, can be attributed mainly to the effect of proton pulses applied during ^15^N variable relaxation delay (T period) to cancel CC. Non-selective hard pulses caused extreme water saturation (80–100 % in most cases), as expected. IBURP-2 pulses, cosine-modulated IBURP-2 pulses, and Watergate-like schemes gave comparable results at slow pulsing rates (R_p_ = 25 s^−1^), with levels of saturation around 10 %, and no significant differences ($\Delta {\text{M}}_{{{\text{sw(800}} - 8 0 )}}^{\text{b/a}}$) between short and long ^15^N relaxation periods.

Faster pulsing rates increase both the level of water saturation and the difference in saturation levels between short and long ^15^N relaxation delays, $\Delta {\text{M}}_{{{\text{sw(800}} - 8 0 )}}^{\text{b/a}}$. At pulsing rates of R_p_ = 200 s^−1^, water saturation was higher for the Watergate scheme than for IBURP-2-based sequences. In addition, a significant increase in the level of saturation was observed between the short and the large relaxation delay when Watergate-like blocks were used to suppress CC ($\Delta {\text{M}}_{{{\text{sw(800}} - 8 0 )}}^{\text{b/a}}$ = 74 % at 600 MHz and 44 % at 800 MHz, see Table 1). In contrast, when using IBURP-2-based sequences, water saturation differences between short and large T periods were less important, around 9–16 %.

These results indicate that the use of an inter-pulse delay of 40 ms (R_p_ = 25 s^−1^) allows the build-up of a steady-state polarization at the start of each scan, in the three approaches tested. Therefore the effect of water saturation is independent of the ^15^N relaxation delay and it should not affect the measured ^15^N R_1_ rates. On the contrary, water saturation increased along this relaxation period when faster pulsing rates were used (R_p_ = 200 s^−1^). Because this effect was stronger for Watergate-like than for IBURP-2-based sequences (IBURP-2 or cosine-modulated IBURP-2 pulses), the latter are expected to show better performance at cancelling CC with minimum water saturation on ^15^N R_1_ measurements.

To study the impact of the previously CC-suppressing schemes on ^15^N longitudinal rates of non-deuterated proteins, eight protocols were compared for the measurement of ^15^N R_1_ at 800 and 600 MHz. They included four proton inversion sequences (Fig. 2 and Fig. S1 SM) at two pulsing rates R_p_ (25 and 200 s^−1^). The individual apparent R_1_ rates obtained at R_p_ = 25 s^−1^ are shown in Fig. 3. The average R_1_, the average pairwise root mean square deviation (*rmsd*) and the correlation coefficients, R, are presented in Table 1 SM.

Measurements carried out at 600 MHz in minimal water saturation conditions (approaches I, II and III) at slow pulsing rates R_p_ = 25 s^−1^ resulted in identical R_1_ rates for methods II (cosine-modulated IBURP-2) and III (Watergate-like pulses), while systematically shorter R_1_ values were obtained for all residues in GB3 when using approach I (IBURP-2), see Fig. 3a. The same effect was observed at 800 MHz (Fig. 3b). The differences in R_1_ values obtained by methods I and II were small (rmsd = 0.075 s^−1^, 3.15 % at 600 MHz and 0.077 s^−1^, 3.76 % at 800 MHz) but systematic and significant, and larger than the reproducibility of the individual experiments (rmsd = 0.030 s^−1^, 1.2 % for duplicate measurements). R_1_ rates measured at 600 MHz using the cosine-modulated IBURP-2 pulses at R_p_ = 25 s^−1^ (approach II) are in perfect agreement (rmsd = 0.022 s^−1^, 0.9 %, Fig. 3d) with those reported for deuterated GB3^1^ at the same field (Lakomek et al. 2012). The same happened for the Watergate-like scheme (Fig. 3e).

The use of hard 180° proton pulses (approach IV), causing strong water saturation, resulted in clear overestimation of R_1_, at both 25 and 200 s^−1^ pulsing rates (Table 1 SM). In addition, errors coming from water saturation effects varied along the protein sequence, being largest in the most solvent-exposed regions of the GB3 protein: residues 15–23 (A region) and 44–55 (B region), Fig. 3a, b. The effect of water saturation can be estimated by the clear correlation between the fractional increase in R_1_ rates, [R_1_(IV) − R_1_(II)]/R_1_(II), and the degree of saturation I_sat_/I_0_ measured in ^1^H–^15^N HSQC spectra (Mori et al. 1995) acquired with and without water presaturation (Fig. S2 SM).

A common alternative to suppress the effect of non-uniform water saturation in R_1_ measurements performed with the classical CC-suppressing scheme employing hard 180° pulses (scheme IV, R_p_ = 200 s^−1^) consists of explicitly purging all proton polarization at the start of the inter-scan delay, using a 90 ^1^H pulse followed by a pulse field gradient. Taking the R_1_ values obtained at 600 MHz using the cosine-modulated IBURP-2 (R_p_ = 25 s^−1^) scheme as reference (approach II) we compared the R_1_ measured by approach IV with and without the purging element. Indeed, the use of the initial purge in scheme IV provides a better agreement with the reference experiment (rmsd = 0.065 s^−1^, 2.6 %, R = 0.931) than approach IV without purging (rmsd = 0.392 s^−1^, 9.3 %, R = 0.195). However such an approach suffers from a significant loss of sensitivity (up to 35 % in the present experiment) and therefore scheme II should be clearly preferred.

Evaluating the effect of R_p_ on R_1_ values measured with approaches II and III, showed that suppression of longitudinal CC at faster proton pulsing rate (R_p_ = 200 s^−1^) resulted in slightly higher R_1_ values for approach II (cosine-modulated IBURP-2), and significantly larger R_1_ rates for approach III (Watergate-like), with respect to those measured at slower pulsing rates (R_p_ = 25 s^−1^). Figures S3 SM and S4 SM show results at 600 and 800 MHz, respectively. R_1_ differences along GB3 amino acid sequence between the two proton pulsing rates were not uniform. Higher deviations appeared for residues 15–23 (A region) and 44–55 (B region), coincident with regions more affected by water saturation effects (approach IV, hard pulses, Fig. 3). This observation is consistent with results shown in Table 1.

Sequence specific R_1_ deviations depend also on the characteristics of the excitation profile of these selective pulses. With cosine-modulated IBURP-2 pulses, showing a wide zero-excitation region around water resonance (Fig. 2), R_1_ differences lower than 3 % were observed in regions A and B. However, the use of Watergate-like pulses yielded R_1_ rates around 6–10 % higher at faster pulsing rates (see Table 2 SM). Degradation of the performance of Watergate-like pulses at high proton pulsing rates may result from overuse of water-selective 90° pulses, continuously exciting water magnetization from +z-axis to the transverse plane and returning it back from +x/+y to +z. Whenever water magnetization is moved from equilibrium it remains in a vulnerable state, due to the strong RD effects, so it is more prone to be in a saturated state.

Our results showed that short pulsing rates resulted in minimal water saturation during the ^15^N relaxation period. It is clear that R_p_ = 25 s^−1^ is enough to cancel longitudinal CC relaxation in GB3 at 600 MHz. For larger non-deuterated proteins, longitudinal CC effects will be smaller because proton spin-flips, resulting from effective proton–proton cross-relaxation, induce exchange between the longitudinal doublet components at a rate faster than their R_1_ difference. The larger the protein, the higher the amide proton spin-flip rate would be. Therefore a pulsing rate of 25 s^−1^ should be high enough to cancel these undesired relaxation mechanisms when measuring ^15^N R_1_ rates at 600 MHz or higher fields on non-deuterated proteins similar to GB3 in size or larger.

Insufficient cancellation of CC with the Watergate approach as a result of both off-resonance effects and pulse imperfection of the central 180° hard proton pulse in the Watergate block has been reported at very high fields (Ishima 2014). With a cosine-modulated IBURP-2 based scheme no 180° hard pulses are involved in CC cancellation. Moreover, larger dispersion in proton spectral widths at higher fields allows the application of these selective pulses at increased offsets from the water resonance, thereby minimizing residual effects of water saturation during the T period. The higher the field, the lower the residual effects would be. Consequently, the use of cosine-modulated IBURP-2 is preferable to Watergate-based schemes in order to obtain more accurate ^15^N R_1_ rates.

### Exploring the effect of saturation of aliphatic protons caused by CC-suppressing IBURP-2-based schemes on ^15^N R_1_ measurements

While R_1_ rates obtained at 600 MHz with the cosine-modulated IBURP-2 approach were very close (Fig. 3d) to those reported for deuterated GB3 at the same field (Lakomek et al. 2012), the amide-selective IBURP-2 scheme gave shorter R_1_ values (Fig. 3c). Systematically shorter rates along the whole GB3 amino acid sequence could not derive from water-saturation effects, because saturated water is very similar for both schemes at R_p_ = 25 s^−1^ (Table 1). The origin of this discrepancy could be inefficient CC suppression or additional saturation effects caused, directly or indirectly, by CC-suppressing pulses, which may affect amide proton magnetization at the beginning of each scan. Differential saturation effects should be sensitive to the recycle delay while cross-correlation should not be affected by this delay.

^15^N R_1_ rates were measured at 600 MHz using IBURP-2 and cosine modulated IBURP-2 with 1.7 and 3.5 s of recycle delay values. While R_1_ rates measured with the second approach were unaffected by recycle delay, R_1_ at long recycle delays using the IBURP-2 approached were larger than values at short delays (Fig. S5 SM). The same happened at 800 MHz. In addition, R_1_ rates at 600 MHz with IBURP-2 pulses and 3.5 s of recycle delay approached to rates reported for deuterated GB3 protein (Lakomek et al. 2012). These results suggested that the observed deviation originates from non-equilibrium magnetization of aliphatic protons caused by the effect of IBURP-2 or cosine modulated IBURP-2 pulses. While aliphatic protons (H^R^) are affected by cosine modulated IBURP-2 pulses, IBURP-2 only excites the amide region (H^N^).

The effect of the CC-suppressing train of pulses will depend on the aliphatic magnetization present at the beginning of the variable ^15^N relaxation period (T). In the pulse sequence shown in Fig. 1 the aliphatic protons magnetization remains on the transverse plane before the T period (XY-pulse program) and is saturated by the PFG G1 applied during the INEPT block. Other pulse sequences used to measure ^15^N R_1_ rates (Lakomek et al. 2012) keep aliphatic protons magnetization aligned along the z-axis (Z-pulse programs) at the start of this period. In both XY- and Z-pulse programs, aliphatic protons magnetization will be affected by CC-suppressing pulses, but in a different way. Thus, depending on the pulse program and on the approach used to cancel CC a T-dependent aliphatic protons saturation level will develop during T.

Of course, the state of non-amide protons magnetization has a significant effect on effective longitudinal relaxation time of amide protons (Pervushin et al. 2002; Schanda 2009), the protons excited at the start of HSQC-based pulse programs to measure ^15^N R_1_ rates. Fast H^N^ relaxation relies on keeping the non-amide magnetization fully aligned along +z, in a non-perturbed state, so saturation of non-amide spins reduce the effective longitudinal H^N^ rates (Schanda 2009). In consequence, slower amide relaxation will take place at high levels of aliphatic spins saturation respect to low saturation states. Slow H^N^ recovery rates will result in a smaller amount of H^N^ magnetization at the start of the pulse program for a fixed recycle delay versus high H^N^ recovery rates. Thus, an increase in H^R^ magnetization saturation at long versus short T periods is expected to produce artificially higher ^15^N R_1_ rates, respect to a T-independent aliphatic protons saturation level. On the contrary, shorter ^15^N R_1_ rates would result from a decreased saturation of aliphatic protons at long respect to short T periods.

We used 1D versions of the pulse programs (XY- and Z) shown in Fig. 4 to measure H^N^ and H^R^ magnetization after a short (80 ms) and a long (800 ms) ^15^N relaxation period, in which IBURP-2 or cosine modulated IBURP-2 sequences were applied to cancel CC. Measurements were performed at 800 MHz. Figure 5 and Table 3 SM show a comparison of the available longitudinal magnetization (non-saturated) after the ^15^N relaxation period (point ii) as a function of the CC-suppression scheme. Also, magnetization was evaluated at point (iii), after a Δ period equivalent to the recycle delay used on the R_1_ measurement experiments, representing the available proton magnetization at the start of the scan. The amount of H^R^ and H^N^ magnetization present after Δ = 1.7 s (recycle delay used on ^15^N R_1_ measurements shown in Table 1 SM) was calculated respect to the magnetization measured with Δ = 3.5 s, and the percentage of H^N^ magnetization is represented in the box at the left over the corresponding 1D spectra (Fig. 5b). In addition, ^15^N R_1_ rates were also measured at 800 MHz with the Z-pulse program using the two CC-suppressing schemes (IBURP-2 and cosine modulated IBURP-2). Average R_1_ rates and the individual apparent R_1_ rates for both XY and Z-pulse programs are presented in Fig. 5a.

With the XY pulse program no signal from aliphatic protons was observed just before the ^15^N relaxation period (point *i* in Fig. 4, Table 3 SM). The longitudinal aliphatic magnetization grows during the ^15^N relaxation time when the aliphatic protons are not excited, this is when CC is suppressed by means of amide-selective IBURP-2 pulses. Because of this, a higher amount of longitudinal H^R^ magnetization has developed at long T periods (800 ms), resulting in a faster recovery of the H^N^ magnetization respect to short T periods (80 ms), see Fig. 5b. A high degree of H^N^ at long periods, at the start of each scan, explains the shorter ^15^N R_1_ rates measured with IBURP-2 (Fig. 5). On the contrary, the train of cosine-modulated IBURP-2 pulses maintains a full saturation of aliphatic protons constant during the T period so the recovery rate of the H^N^ magnetization is unaffected. Thus, ^15^N R_1_ relaxation rates measured with this scheme are not subject to saturation effects.

On the other hand, when the aliphatic protons magnetization is placed on the z-axis at the beginning of the ^15^N relaxation T period (Z pulse program), cosine-modulated IBURP-2 pulses cause T period-dependent variable degrees of saturation, the larger the relaxation period the larger the saturation level (Fig. 5b). As a consequence, the amount of H^N^ polarization at the beginning of each scan is reduced for long T values, resulting in artificial and significant increased ^15^N R_1_ rates (Fig S6 SM). The use of amide-selective IBURP-2 pulses to cancel CC, in principle, should not affect the aliphatic protons as their magnetization is not directly perturbed. However, amide protons inversion during the train of IBURP-2 pulses perturbs aliphatic protons magnetization by cross-relaxation and the non-equilibrium aliphatic magnetization eventually feeds-back to the amide protons in a way that depends on the ^15^N relaxation delay. Actually, a slightly higher degree of aliphatic protons saturation is observed at long T periods resulting in a reduced amount of H^N^ magnetization at the start of the scan, versus that observed at short T periods. This is consistent with an erroneous increase of measured ^15^N R_1_ rates with this scheme.

## Concluding remarks

Systematic errors in ^15^N R_1_ measurements arise from unwanted saturation of water and aliphatic protons by the train of 180° proton pulses used to eliminate cross-correlations effects. Errors coming from water saturation show a high degree of variability, being more important for residues in solvent-exposed regions of the protein, whose amide protons show fast exchange with water.

We have shown that saturation of aliphatic protons is another important source of systematic errors on ^15^N R_1_ rates measurements in non-deuterated proteins. Errors show less variability along the amino acid sequence, although they are probably sensitive to local variations in the relaxation rates of relevant protons.

Maintaining the transverse magnetization of aliphatic protons in a dephased state (saturated state) during the variable ^15^N relaxation period T of the XY-pulse scheme by using the cosine modulated IBURP-2 scheme ensures that the amount of H^N^ polarization at the start of the pulse sequence does not depend on the length of the variable relaxation delay T.

In conclusion, the optimized pulse scheme shown in Fig. 1 (XY-sequence) using cosine-modulated IBURP-2 pulses spaced 40 ms minimize systematic errors on ^15^N R_1_ rates measurements in non-deuterated proteins. This sequence efficiently controls both water and aliphatic protons saturation while cancelling CC effects during the variable ^15^N relaxation period. The optimized pulse program applied to protonated GB3 gives identical ^15^N R_1_ values to those obtained with a deuterated sample.

## Electronic supplementary material

Supplementary material 1 (PDF 722 kb)

## Footnotes

1 The effect of deuteration of non-exchanging protons on ^15^N R_1_ rates is expected to be insignificant for a protein the size of GB3. Thus ^15^N R_1_ for a protonated sample of GB3 and for a deuterated one should be very similar. In addition, R_2_ rates measured at 600 MHz with non-deuterated GB3 were also coincident with those obtained for deuterated GB3 (Lakomek et al. 2012); rmsd = 0.072 s^−1^ (1.28 %).
