# Supplementary material for: An optimized method for 15N R1 relaxation rate measurements in non-deuterated proteins
Source: J Biomol NMR. 2015 May 7;62(2):209–20. doi: 10.1007/s10858-015-9937-4 (PMC4451471; doi:10.1007/s10858-015-9937-4)
Supplement: Supplementary file 1 — Supplementary material 1 (PDF 722 kb) [file 10858_2015_9937_MOESM1_ESM.pdf]

Supplementary Material

An optimized method for ^{15}N R_1 relaxation rate measurements in non-deuterated proteins

Margarida Gairí,[a,*] Andrey Dyachenko,[b] M. Teresa González,[a] Miguel Feliz,[a] Miquel Pons,[c] Ernest Giralt,[b,*]

[a] NMR Facility. Scientific and Technological Centers University of Barcelona (CCiTUB), Baldiri Reixac 10, 08028-Barcelona (Spain)

[b] Institute for Research in Biomedicine (IRB), Baldiri Reixac 10, 08028-Barcelona (Spain)

[c] Biomolecular NMR Laboratory & Organic Chemistry Department, University of Barcelona, Baldiri Reixac 10, 08028-Barcelona (Spain)

*Corresponding authors:

M. Gairí, NMR Facility. Scientific and Technological Centers University of Barcelona (CCiTUB), Baldiri Reixac 10, 08028-Barcelona, e-mail: mgairi@rmn.ub.edu, phone: +34 93 4034466, fax: +34 93 4034465

E. Giralt, Institute for Research in Biomedicine (IRB), Baldiri Reixac 10, 08028-Barcelona, e-mail: ernest.giralt@irbbarcelona.org, phone:+34 93 4037125, fax: +34 93 4037126

Figures

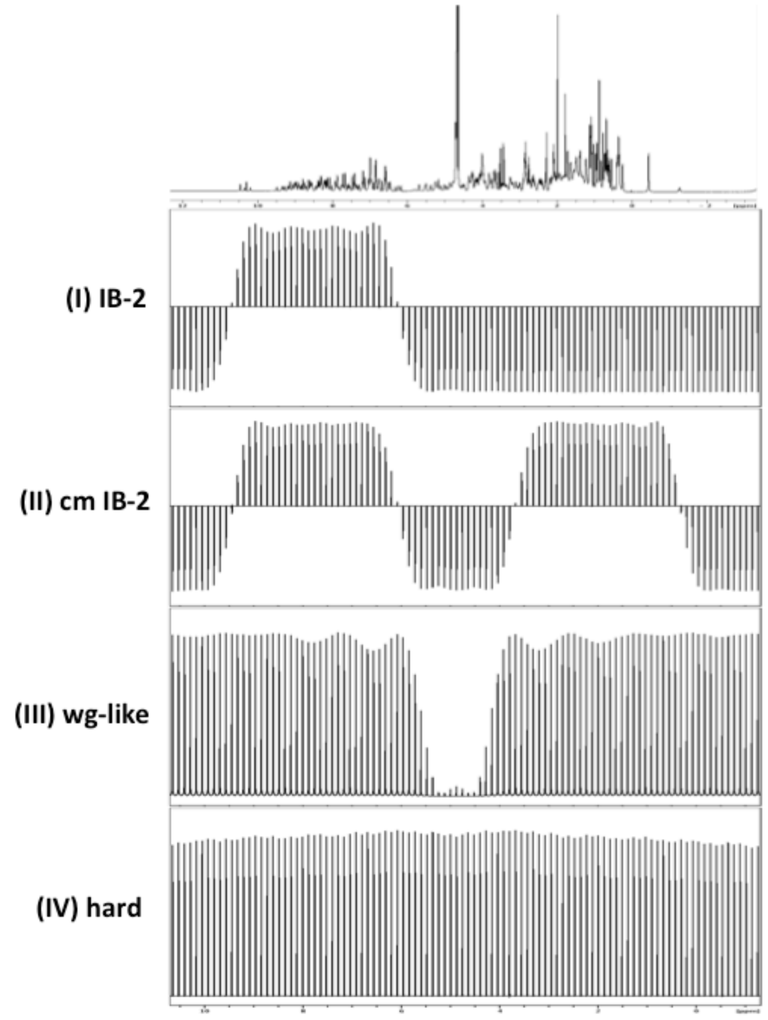

Figure S1. ^1H spectrum of ^{15}N-GB3 and experimental excitation profiles at 600 MHz for several types of proton inversion elements used to cancel CC during ^{15}N relaxation period T: **(I) IB-2**: amide-selective IBURP-2 pulse (1.9 ms, offset 2400 Hz from water frequency); **(II) cm IB-2**: cosine-modulated IBURP-2 pulse (1.9 ms, offset ± 2400 Hz from water frequency); **(III) wg-like**: watergate-like pulses: water-selective $90°_{-x}$ pulse (1 ms, Square) – hard $180°_x$ pulse (21 µs) – water-selective $90°_{-x}$ pulse (1 ms, Square); **(IV) hard**: non-selective hard 180° pulse (21 µs). Excitation profiles for I, II and IV were measured with a pulse program consisting of the selective 180° pulse, followed by a pulsed field gradient and a final short readout pulse, while profile for III was obtained using a pulse program consisting of an initial 90° pulse, followed by a $90°_{sel}$-$180°_{hard}$-$90°_{sel}$ block inserted in a pulsed field gradient echo and a final short readout pulse. Each spectrum was acquired with a single scan and the offsets vary along 10000 Hz in 100 Hz-steps. A $CuSO_4$-doped water sample in D_2O was used.

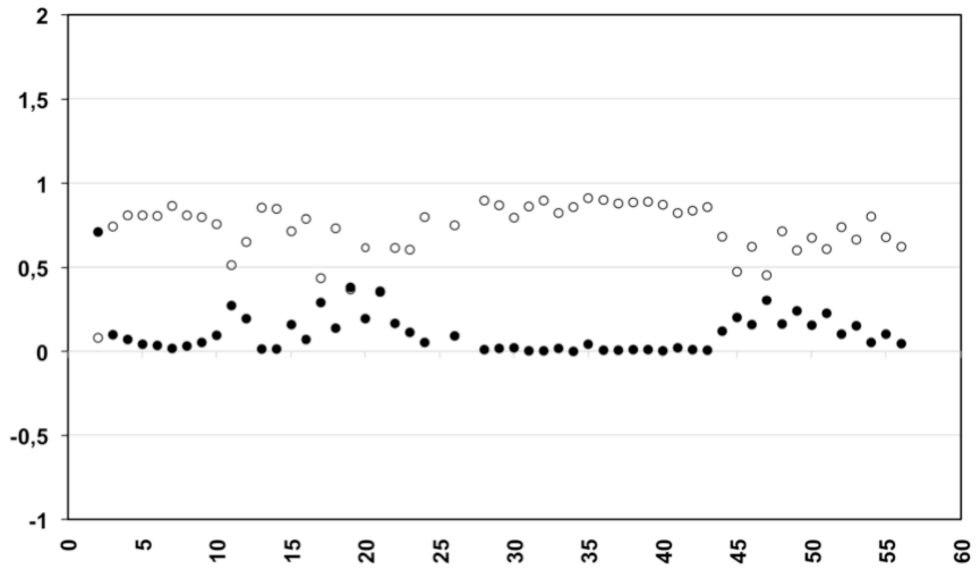

Figure S2. Fractional increase on ^{15}N R_1 rates, $[R_1(IV)-R_1(II)]/R_1(II)$, at 800 MHz, along GB3 aa sequence, is represented with black spheres. R_1 (IV) were measured with hard 180° proton pulses to cancel CC during the ^{15}N relaxation period T, while cosine-modulated IBURP-2 180° proton pulses were used on R_1 (II) measurements. In both cases R_p=25 s^{-1}. The degree of saturation I_{sat}/I_0, represented with open spheres, was obtained by comparing amide signal intensities resulting from two ^1H-^{15}N HSQC experiments, one acquired with water presaturation during relaxation delay (I_{sat}) and one measured without presaturation (I_0). Then, amide residues less affected by exchange saturation transfer from water protons are those showing I_{sat}/I_0 close to 1, while this value significantly deviates from 1 when saturation transfer is important, the stronger the effect the lower the I_{sat}/I_0 value. A clear correlation is observed between residues yielding lower I_{sat}/I_0 ratios and those presenting significant R_1 deviations.

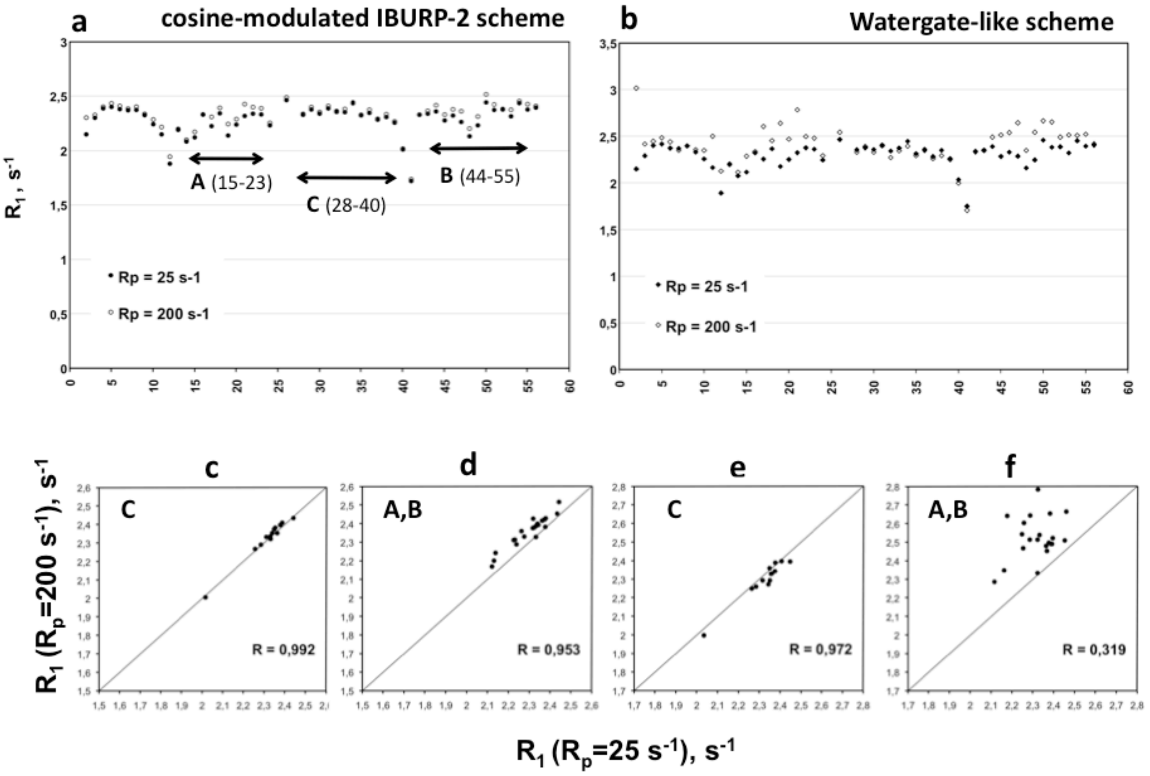

Figure S3. Effect of proton pulsing rate R_p to cancel CC during ^{15}N relaxation delay T, on R_1 relaxation rates measured for non-deuterated ^{15}N-GB3 at 600 MHz. **a,** R_1 values at slow (25 s^{-1}) and fast (200 s^{-1}) proton pulsing rates obtained with cosine-modulated IBURP-2 pulses (II); **b,** R_1 values at slow (25 s^{-1}) and fast (200 s^{-1}) proton pulsing rates measured using Watergate-like pulses (III); **c-d,** correlation plots of R_1 rates measured with cosine-modulated IBURP-2 pulses applied at R_p=25 s^{-1} and R_p=200 s^{-1} for several protein regions: C region (residues 28-40), A,B regions (residues 15-23 and residues 44-55); **e-f,** correlation plots of R_1 rates measured with Watergate-like pulses applied at R_p=25 s^{-1} and R_p=200 s^{-1} for C region (residues 28-40) and for A,B regions (residues 15-23 and residues 44-55).

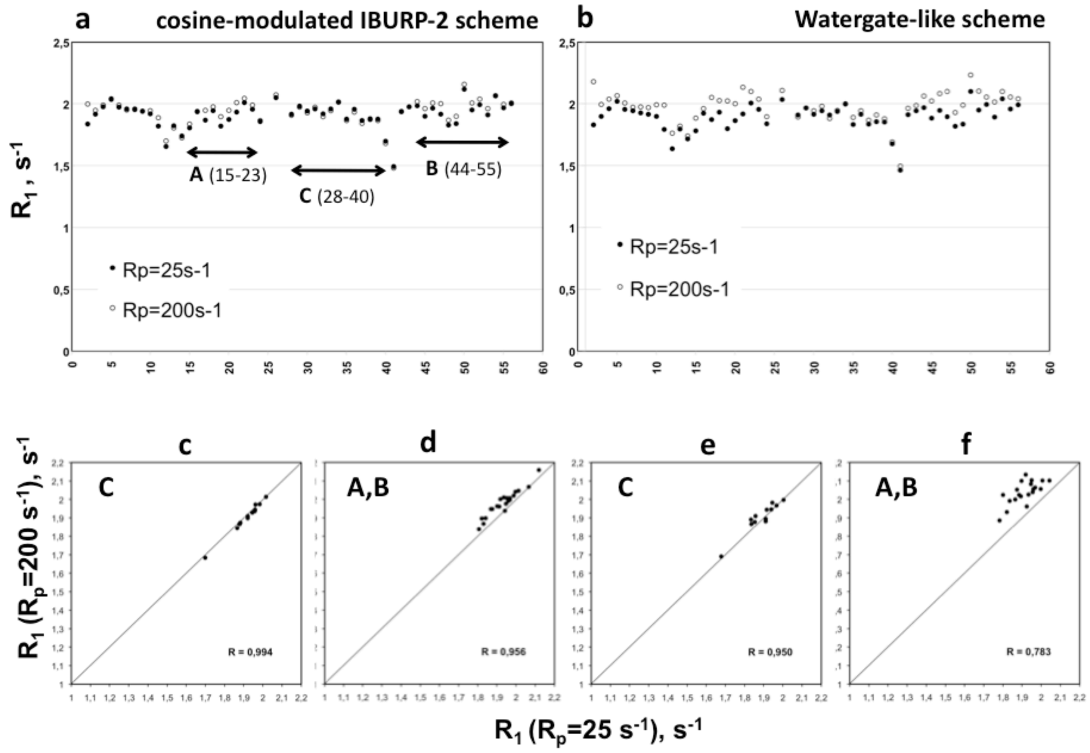

Figure S4. Effect of proton pulsing rate R_p to cancel CC during ^{15}N relaxation delay T, on R_1 relaxation rates measured for non-deuterated ^{15}N-GB3 at 800 MHz. **a,** R_1 values at slow (25 s^{-1}) and fast (200 s^{-1}) proton pulsing rates obtained with cosine-modulated IBURP-2 pulses (II); **b,** R_1 values at slow (25 s^{-1}) and fast (200 s^{-1}) proton pulsing rates measured using watergate-like pulses (III); **c-d,** correlation plots of R_1 rates measured with cosine-modulated IBURP-2 pulses applied at R_p=25 s^{-1} and R_p=200 s^{-1} for several protein regions: C region (residues 28-40), A,B regions (residues 15-23 and residues 44-55); **e-f,** correlation plots of R_1 rates measured with watergate-like pulses applied at R_p=25 s^{-1} and R_p=200 s^{-1} for C region (residues 28-40) and for A,B regions (residues 15-23 and residues 44-55).

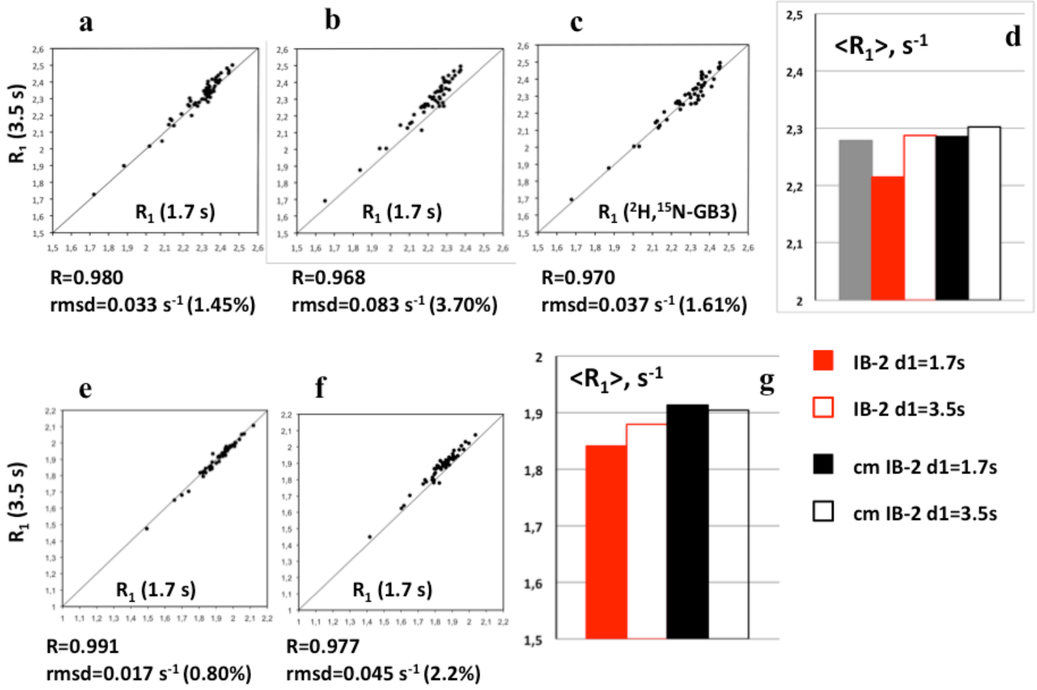

Figure S5. Effect of recycle delay (d1) on R_1 relaxation rates measured for non-deuterated ^{15}N-GB3 using cosine-modulated IBURP-2 or amide-selective IBURP-2 pulses (R_p=25 s^{-1}) at 600 MHz (**a-d**) and 800 MHz (**e-g**). **a,** correlation plots of R_1 rates measured with cosine-modulated IBURP-2 pulses at 1.7 s and 3.5 s of recycle delay; **b,** correlation plots of R_1 rates measured with amide-selective IBURP-2 pulses at 1.7 s and 3.5 s of recycle delay; **c,** correlation plots of R_1 rates measured with deuterated GB3 (Lakomek and Bax 2012) and with non-deuterated GB3 using IBURP-2 pulses at a recyle delay of 3.5 s; **d,** average R_1 relaxation rates at 600 MHz (the column in gray corresponds to the average R_1 rate for deuterated GB3 protein, reported by Lakomek and Bax, 2012); **e,** correlation plots of R_1 rates measured with cosine-modulated IBURP-2 pulses at 1.7 s and 3.5 s of recycle delay; **f,** correlation plots of R_1 rates measured with amide-selective IBURP-2 pulses at 1.7 s and 3.5 s of recycle delay; **g,** average R_1 relaxation rates at 800 MHz

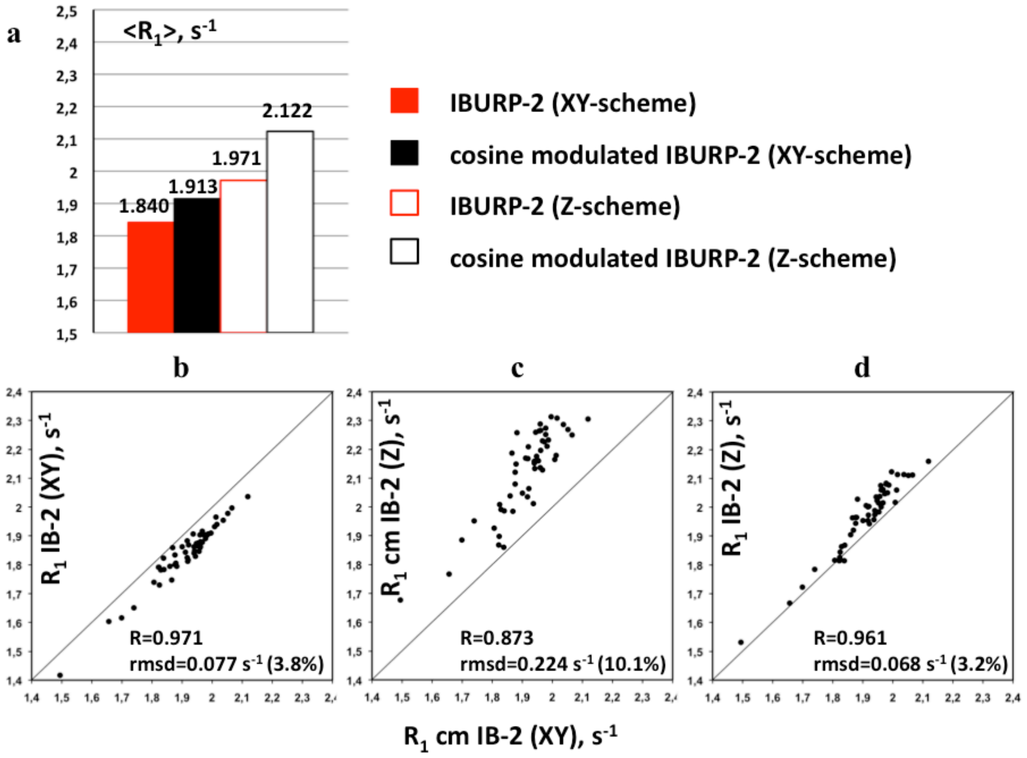

Figure S6. a, Average ^{15}N R_1 relaxation rates measured at 800 MHz for non-deuterated ^{15}N-GB3 using XY- and Z-pulse schemes (see Fig. 4), and CC-suppressing schemes based on amide-selective IBURP-2 pulses or cosine-modulated IBURP-2 pulses (R_p=25 s^{-1}), applied during the variable ^{15}N relaxation delay (T). **b,** correlation plot of R_1 rates measured using the XY-pulse sequence with cosine modulated IBURP-2 pulses and IBURP-2 pulses; **c,** correlation plot of R_1 rates measured using cosine-modulated IBURP-2 pulses to cancel CC, with the XY-sequence and the Z-sequence; **d,** correlation plot of R_1 rates measured with the XY-pulse program using cosine-modulated IBURP-2 pulses and with the Z-pulse sequence using IBURP-2 pulses.

Tables

Comparison of ^{15}N R_1 rates measured at 600 MHz						
	$R_p = 25\ s^{-1}$			***$R_p = 200\ s^{-1}$***		
	average R_1 (s^{-1})	***rmsd [a] s^{-1} (%)***	***R [b]***	***average R_1 (s^{-1})***	***rmsd [a] s^{-1} (%)***	***R [b]***
(I) IB-2	2.215	0.075 (3.15%)	0.986	2.255	0.071 (2.90%)	0.990
**(II) cm IB-2**	**2.286**	**0**	**1**	**2.323**	**0**	**1**
(III) wg-like	2.298	0.018 (0.74%)	0.995	2.411	0.166 (5.48%)	0.686
(IV) hard	2.523	0.428 (10.23%)	0.156	2.485	0.349 (8.32%)	0.398
Comparison of ^{15}N R_1 rates measured at 800 MHz						
	$R_p = 25\ s^{-1}$			***$R_p = 200\ s^{-1}$***		
	average R_1 (s^{-1})	***rmsd [a] s^{-1} (%)***	***R [b]***	***average R_1 (s^{-1})***	***rmsd [a] s^{-1} (%)***	***R [b]***
(I) IB-2	1.841	0.077 (3.76%)	0.971	1.874	0.067 (3.22%)	0.968
**(II) cm IB-2**	**1.913**	**0**	**1**	**1.935**	**0**	**1**
(III) wg-like	1.895	0.020 (0.97%)	0.996	1.977	0.052 (2.34%)	0.950
(IV) hard	2.116	0.266 (8.46%)	0.331	2.215	0.353 (8.58%)	0.480

Table 1 SM Comparison of ^{15}N R_1 rates measurements in non-deuterated GB3, performed with different CC- suppressing conditions during the variable ^{15}N relaxation period T. [a]Average pairwise root mean square deviations (rmsd) resulting from comparison of rates measured with schemes (I), (III) and (IV) relative to rates obtained with scheme (II). [b]Correlation coefficients (R) of R_1 are calculated for schemes (I), (III) and (IV) relative to (II). Comparison is done independently for each proton pulsing rate R_p and for each magnetic field. Average rmsd values for identical R_1 measurements performed to test experimental reproducibility were lower than 0.030 s^{-1} (1.2%).

Effect of R_p on R_1 rates at 600 MHz								
Regions	***(II) cm IB-2***				***(III) wg-like***			
	Average R_1 (s^{-1})		***rmsd[a]***	***R^b***	***Average R_1 (s^{-1})***		***rmsd***	***R***
	**25 s^{-1}**	**200 s^{-1}**	***s^{-1} (%)***		**25 s^{-1}**	**200 s^{-1}**	***s^{-1} (%)***	
All [c]	2.286	2.323	0.050 (2.0%)	0.970	2.298	2.411	0.203 (8.2%)	0.516
C [d]	2.318	2.329	0.017 (0.7%)	0.992	2.328	2.301	0.035 (1.4%)	0.972
A+B [e]	2.296	2.355	0.065 (2.7%)	0.953	2.313	2.521	0.239 (9.7%)	0.319

Effect of R_p on R_1 rates at 800 MHz								
Regions	***(II) cm IB-2***				***(III) wg-like***			
	Average R_1 (s^{-1})		***rmsd[a]***	***R^b***	***Average R_1 (s^{-1})***		***rmsd***	***R***
	**25 s^{-1}**	**200 s^{-1}**	***s^{-1} (%)***		**25 s^{-1}**	**200 s^{-1}**	***s^{-1} (%)***	
All [c]	1.913	1.935	0.042 (2.0%)	0.943	1.895	1.977	0.108 (5.1%)	0.821
C [d]	1.912	1.901	0.014 (0.7%)	0.994	1.890	1.907	0.030 (1.5%)	0.950
A+B [e]	1.931	1.977	0.052 (2.4%)	0.956	1.918	2.043	0.134 (6.4%)	0.783

Table 2 SM Effect of proton pulsing rate (R_p) to cancel CC during the variable ^{15}N relaxation delay T on ^{15}N R_1 rates measured in non-deuterated GB3. [a]Average pairwise root mean square differences resulting from comparison of R_1 rates at R_p=25 s^{-1} and R_p =200 s^{-1}; [b]Correlation coefficients of R_1 values calculated at R_p=25 s^{-1} and R_p=200 s^{-1}; [c]R_1 average values, rmsd and R are calculated considering all residues of GB3 amino acid sequence; [d] only residues 28-40 (C region) are considered; [e] only residues 15-23 (A region) and residues 44-55 (B region) are considered (see Fig. S3 and S4)

	**point (i) (before T)**		**CC scheme**	**T period**	**point (ii) (after T)**		**Point (iii) Δ = 1.7 s**	
	H^R	H^N			H^R	H^N	H^R	H^N
XY-pulse program	0%	0%	IB-2	80 ms	9%	0%	89%	90%
				800 ms	57%	11%	92%	94%
			cos mod IB-2	80 ms	0%	3%	88%	85%
				800 ms	0%	3%	88%	85%
Z-pulse program	76%	0%	IB-2	80 ms	80%	5%	94%	93%
				800 ms	76%	1%	93%	90%
			cos mod IB-2	80 ms	52%	10%	93%	91%
				800 ms	3%	0%	88%	87%

Table 3 SM Evaluation of the amount of aliphatic protons (H^R) and amide protons (H^N) magnetization present at points (i), (ii) and (iii) of the XY- and Z-pulse programs shown in Fig. 4 of the manuscript. ^{1}H spectra of GB3 were measured at 800 MHz just before the 15N relaxation period T (point i), immediately after the application of a train of amide-selective IBURP-2 pulses or cosine-modulated IBURP-2 pulses (with 40 ms inter-pulse spacing) for relaxation periods T of 80 ms and 800 ms (point ii), and also after a delay Δ=1.7s to evaluate magnetization present at the start of the sequence (point iii). The aliphatic region between 2.25 ppm and 0.25 ppm and the amide region between 9.55 ppm and 7.25 ppm are integrated in each spectrum respect to the amount of the equilibrium H^R or H^N polarization, respectively, measured at point (iii) for Δ=3.5 s in the corresponding sequence.
